# Anti-Müllerian Hormone Signal Transduction involved in Müllerian Duct Regression

**DOI:** 10.3389/fendo.2022.905324

**Published:** 2022-06-02

**Authors:** Richard L. Cate

**Affiliations:** Department of Chemistry, Boston University, Boston, MA, United States

**Keywords:** anti-müllerian hormone, transforming growth factor-β, bone morphogenetic protein, Müllerian duct regression, signal transduction

## Abstract

Over seventy years ago it was proposed that the fetal testis produces a hormone distinct from testosterone that is required for complete male sexual development. At the time the hormone had not yet been identified but was invoked by Alfred Jost to explain why the Müllerian duct, which develops into the female reproductive tract, regresses in the male fetus. That hormone, anti-Müllerian hormone (AMH), and its specific receptor, AMHR2, have now been extensively characterized and belong to the transforming growth factor-β families of protein ligands and receptors involved in growth and differentiation. Much is now known about the downstream events set in motion after AMH engages AMHR2 at the surface of specific Müllerian duct cells and initiates a cascade of molecular interactions that ultimately terminate in the nucleus as activated transcription factors. The signals generated by the AMH signaling pathway are then integrated with signals coming from other pathways and culminate in a complex gene regulatory program that redirects cellular functions and fates and leads to Müllerian duct regression.

## Introduction

Anti-Müllerian hormone (AMH), also called Müllerian inhibiting substance (MIS), is a member of the transforming growth factor-β (TGF-β) family expressed in Sertoli cells of the fetal and postnatal testis and granulosa cells of the postnatal ovary and has important roles in male and female reproductive development (1). In the male vertebrate embryo, AMH is responsible for the regression of the Müllerian duct (MD), the anlagen of the uterus, Fallopian tubes, and upper part of the vagina (2). Testosterone is produced later in male sexual development and is responsible for differentiation of the epididymis, vas deferens, and seminal vesicles from the Wolffian duct. However, sexual development of the male is not complete unless AMH causes regression of the MD. In the adult male, AMH has a role in Sertoli and Leydig cell differentiation and function (3, 4). In females, AMH inhibits primordial follicle recruitment and the responsiveness of growing follicles to follicle-stimulating hormone (5). In addition, AMH has been reported to have roles within the nervous system (6, 7) and the hypothalamic-pituitary-gonadal axis (8, 9).

Over 33 TGF-β ligands have been identified. AMH is closer in lineage and function to a group of TGF-β family members, which includes the bone morphogenetic proteins (BMPs), growth differentiation factors (GDFs), and activins. These proteins have important roles in embryonic patterning and morphogenesis, as well as more specialized roles within organs, such as the control of gonadal function and the regulation of bone and muscle mass (10). Another branch of the family, the TGF-βs, appeared later in evolution with the vertebrates and regulate more newly acquired processes including injury repair, cellular proliferation, adhesion, and immunity (11). TGF-β ligands mediate their effects by assembling a transmembrane receptor complex of type I and type II components, both of which contain intracellular serine/threonine kinase domains. The close juxtaposition of the two receptors results in the phosphorylation and activation of the type I receptor kinase by the constitutively active kinase domain of the type II receptor (12–14). Once activated, the type I receptor phosphorylates a receptor-regulated Smad (R-Smad), which interacts with the Co-mediator Smad4 to form a complex. This complex then moves to the nucleus where it associates with other transcription factors and binds to a Smad-binding element (SBE) in the promoter or enhancers of target genes (15). Five type II receptors (TGFR2, ActR2, ActR2B, BMPR2, and AMHR2), seven type I receptors (ALK1-7), and five R-Smads (1-3, 5, and 8) have been identified. There are two Smad pathways: TGF-β, activins and some GDFs stimulate the phosphorylation of Smad2 and Smad3, whereas BMPs and some GDFs stimulate the phosphorylation of Smad1, 5, and 8. Due to the limited number of type I and II receptors, each receptor interacts with multiple ligands, and many ligands interact with multiple receptors. Uniquely for the family, AMH and its type II receptor AMHR2 are mutually specific, as indicated by the identical phenotypes of *Amh* and *Amhr2* null mice (16). However, AMH does share the type I receptors, ALK2, ALK3, and ALK6, and the Smads 1, 5, and 8 with the BMP pathway (17).

**Figure 1:**
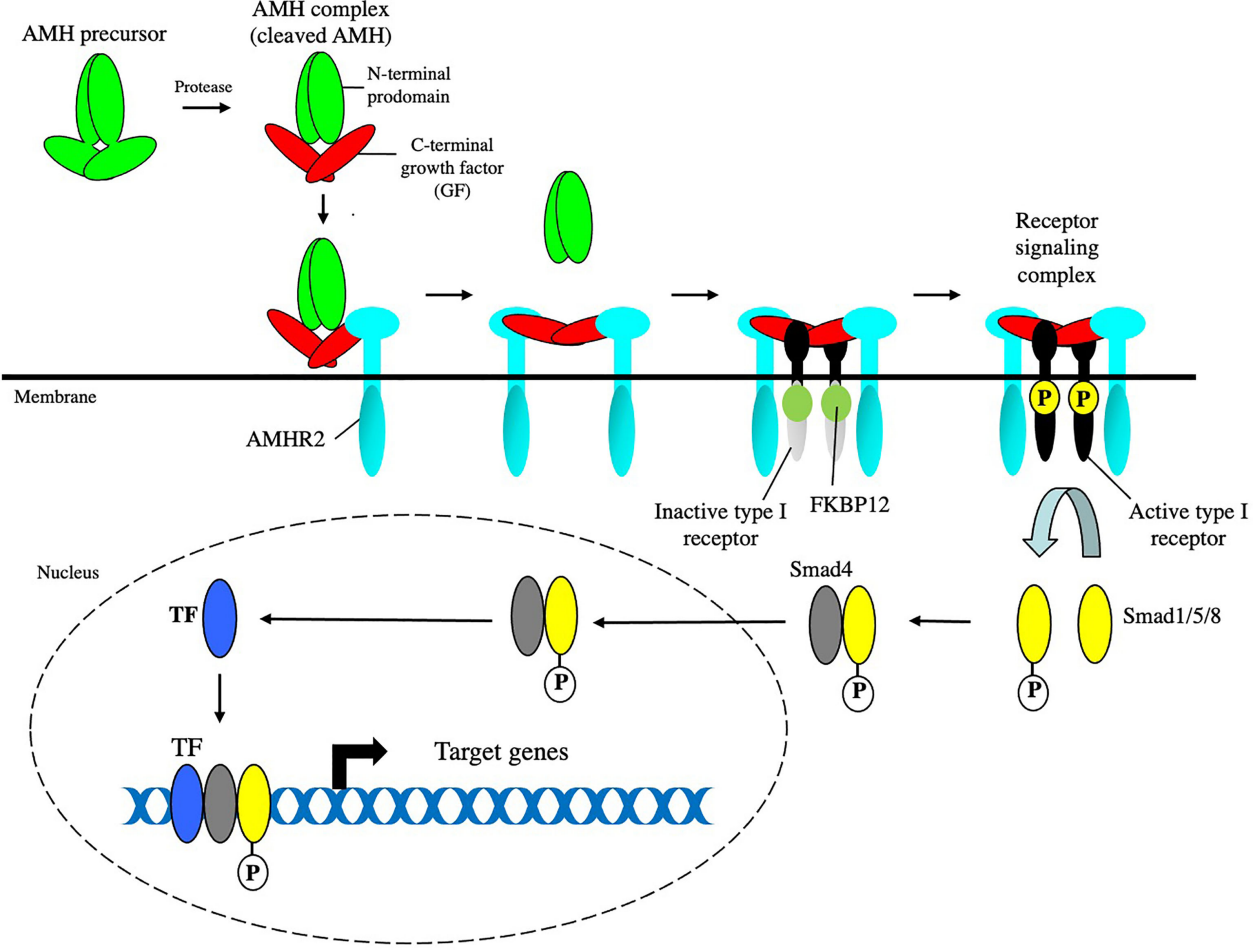
The canonical AMH signaling pathway. AMH assembles a receptor signaling complex composed of type I and type II receptors. The kinase domain of AMHR2 is constitutively active and phosphorylates and activates the kinase domain of the type I receptor (ALK2 or 3), which phosphorylates Smad1, 5, and 8. The phosphorylated Smads form a heterotrimer with Smad4, which translocates to the nucleus, and binds to other transcription factors (TF). The Smad/TF complex binds to promoter or enhancer regions of target genes to regulate transcription.

Regression of the MD induced by AMH in the male fetus, proceeds *via* a paracrine mechanism, since it is the surrounding mesenchymal cells that express AMHR2 (18, 19). Thus, AMH signaling either activates a paracrine factor leading to apoptosis of the epithelium, or alternatively, represses a survival signal required for epithelial cell growth (20). Breakdown of the basement membrane (21–23), proteolysis (20), epithelial cell migration (24), and apoptosis and epithelial to mesenchymal cell transformation (25), have been shown to play a role in regression. This review will cover what is now known about the canonical AMH signaling pathway (**Figure 1**) and place it in context with other signaling pathways that are required for MD regression.

## The Canonical Amh Signaling Pathway

### AMH

**Figure 2:**
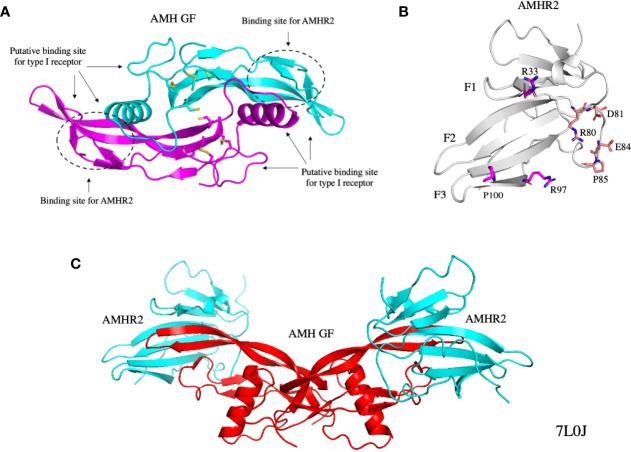
Structures of the AMH GF **(A)**, AMHR2 ECD **(B)**, and the AMH GF bound to AMHR2 ECDs **(C)**. The binding sites of the type I receptor and AMHR2 on the AMH GF are indicated and some of the residues on the AMHR2 ECD that contact the AMH GF are shown as sticks. All structures were generated from PDB 7L0J (29). GF, growth factor; ECD, extracellular domain.

The human *AMH* gene is composed of five exons, maps to chromosome 19 p13.3 (26), and encodes a protein of 560 amino acids containing a 24 amino acid signal sequence. The bovine testicular (27) and human recombinant (28) proteins are synthesized as homodimeric precursors, containing an N-terminal prodomain and a smaller C-terminal mature domain. The C-terminal domain, herein referred to as the growth factor (GF), contains a cysteine knot motif that is characteristic of all TGF-β family members, and has binding sites for AMHR2 and the type I receptor (29) (**Figure 2A**) (29, 30). Cleavage of the precursor at monobasic sites between the two domains is required for binding to AMHR2, but after cleavage the prodomain and C-terminal homodimers remain associated in a noncovalent complex (28). The prodomain has been shown to play an important role in the folding of the GF and the secretion of the AMH precursor and noncovalent complex (31).

Only ~5% of AMH secreted from bovine testis and Chinese hamster ovary cells is cleaved at the monobasic sites, but complete processing can be achieved *in vitro* with plasmin (28). The kex2/subtilisin-like endoprotease PCSK5 may be responsible for cleavage *in vivo* (32). Variations in the level of proteolytic cleavage of AMH have been observed in body fluids during various developmental and metabolic conditions (33, 34). A higher level of cleavage was observed in the follicular fluid from females with polycystic ovary syndrome (PCOS), consistent with an autocrine role for AMH in the pathophysiology of PCOS in the follicle (35). However, while most of the cleaved AMH in the follicular fluid was competent for binding AMHR2, only a small fraction of the cleaved AMH in serum was found to be competent (35). These results indicate that AMH can be subjected to further structural changes after cleavage that prevent binding to AMHR2 and render it inactive.

AMH is expressed by Sertoli cells of the testes, starting eight weeks after fertilization in humans (36), and persisting at high levels until puberty, when they decrease to below detection in the adult (37). In the female, AMH is expressed in granulosa cells of the ovary, starting 23 weeks after fertilization with the appearance of primary follicles (38); the highest expression levels are in secondary and small antral follicles (39). AMH can be detected in the blood until the onset of menopause, when levels fall below detection, concomitant with the decrease in the number of developing follicles (40).

### AMHR2

The human *AMHR2* gene is composed of 11 exons, maps to chromosome 12q13 (41), and encodes a 573 amino acid membrane protein containing a signal sequence, an N-terminal extracellular domain that binds AMH, a single transmembrane domain, and an intracellular domain with serine/threonine kinase activity. AMHR2 uses its transmembrane domain for insertion and orientation in the membrane, because its signal sequence is not functional (42). The extracellular domain (**Figure 2B**) has the structure of a three-finger toxin found in other TGF-β type II receptors (29), and four similar disulfide bridges. The intracellular domain has the structure of a two-domain kinase, as deduced from a molecular model (42). During biosynthesis of AMHR2, aberrant cleavage of the extracellular domain and the formation of promiscuous disulfide bonds can occur, leading to intracellular retention (43). In the absence of ligand, AMHR2 undergoes a high level of non-covalent homo-oligomerization at the plasma membrane (43), even higher than the levels observed for TGFR2 and BMPR2 (44).

AMHR2 is expressed in the MDs of both sexes by mesenchymal cells surrounding the epithelium (18, 19) according to a cranio-caudal gradient (25), and disappears in the male after MD regression. AMHR2 is also expressed in the male and female gonads. In the testis, it is expressed from fetal life to puberty, whereas in the ovary, AMHR2 is expressed from fetal life to adulthood by granulosa cells of preantral and antral follicles (45). In humans, mutations in the genes coding for *AMH* or *AMHR2 cause* persistent Müllerian duct syndrome (PMDS), in which normal 46,XY males retain a uterus and fallopian tubes (46, 47). Many of these mutations lead to unstable and/or truncated proteins. Recently, loss of function heterozygous mutations in *AMH* and *AMHR2* were identified in a small subset of patients with congenital hypogonadotropic hypogonadism (48). Mutations in *AMH* and *AMHR2* produce a spectrum of phenotypes in a variety of vertebrate species (49).

### Binding of the noncovalent AMH complex to AMHR2

**Figure 3:**
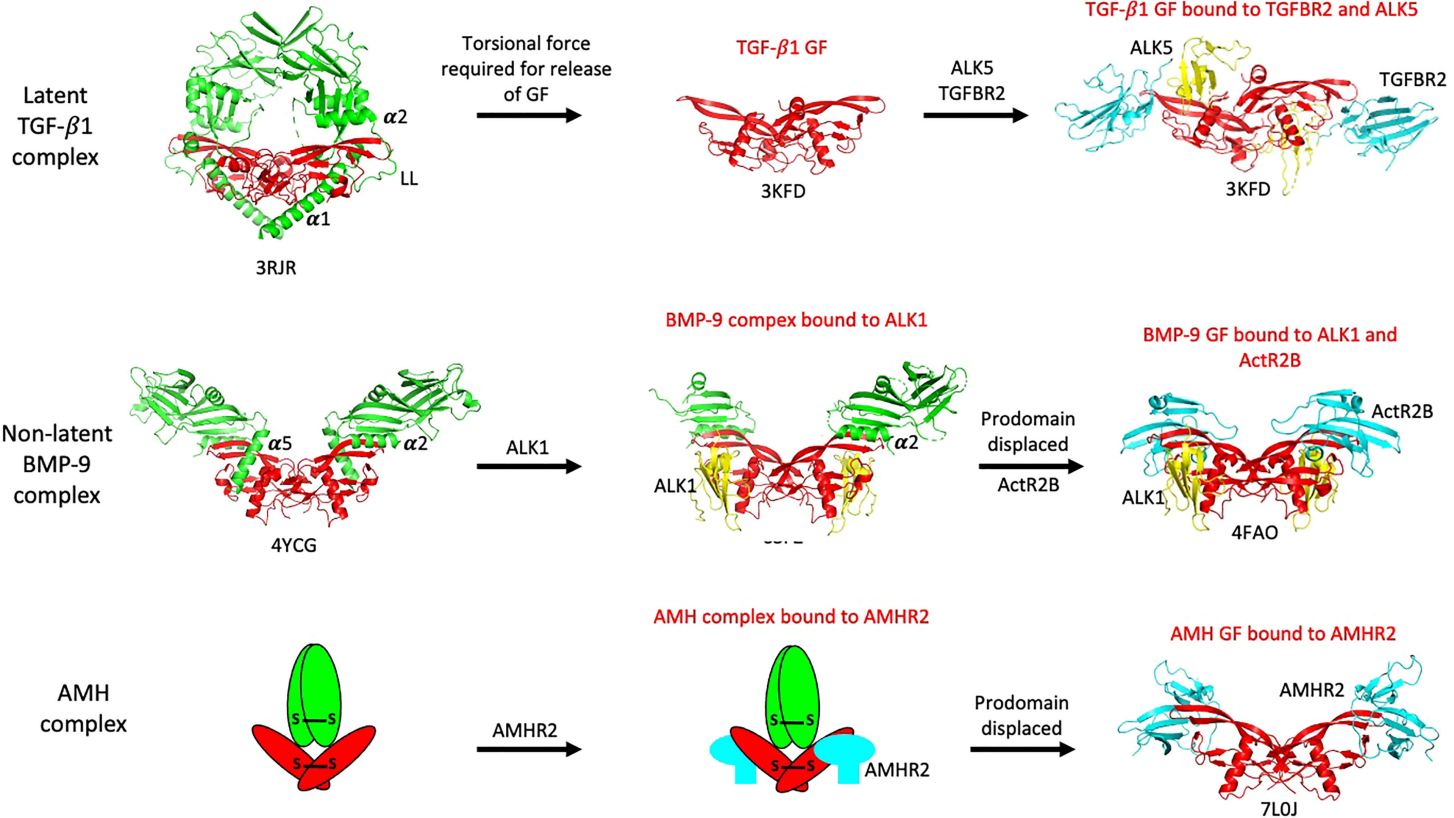
Summary of how various TGF-β family ligands access their receptors. The latent TGF-β1 complex requires torsional force to liberate its GF and allow access to receptors. The non-latent BMP-9 complex can bind to its type I receptor, ALK1, without inducing prodomain displacement, while the AMH complex can bind to AMHR2 in solution without inducing prodomain displacement. Prodomains are shown in green, GFs in red, type I receptor ECDs in yellow, and type II receptor ECDs in cyan. The PDB file names are shown beneath each structure. The α1-, α2-, and α5-helices and latency lasso (LL) in the prodomains are labeled; these elements are located in close proximity to receptor binding sites on the GFs.

An early question that arose prior to the identification of AMHR2, was whether the prodomain of AMH would prevent binding of the AMH GF to its receptor. Whereas the noncovalent complexes of some TGF-β ligands are latent and cannot bind their receptors, the noncovalent complexes of other ligands are biologically active, indicating that they can bind their receptors. Crystal structures of noncovalent complexes of TGF-β ligands (50–53) have provided insight into the way that prodomains interact with their GFs, revealing that the latency lasso, α2-helix, and β1-strand of the prodomain form proximal associations with the type II receptor binding site on the GF, while the α1- or α5-helix forms a proximal association with the type I receptor binding site (14). Still, it has not been determined whether these associations block access to receptors. The GFs of latent complexes are bound to their prodomains in a more confined state and either torsional force (TGF-β1 (50);) or proteolysis (GDF-8 (53);) is required for the GF to be released from their prodomains and allow receptor interactions. In contrast, non-latent complexes can bind their receptors while the prodomain is still bound to the GF. For example, the BMP-9 complex can bind to the type I receptor ALK1 (54) even though the α5-helix of the prodomain is associated with the type I receptor binding site in the noncovalent complex (51). The differences in how the latent TGF-β1 and non-latent BMP-9 complexes access their receptors are illuminated in **Figure 3**. The regions in the AMH prodomain that correspond to the β1-strand and α2-helix elements in TGF-β1 and BMP-9 are not well conserved (51), so structural verification will be required to determine whether the AMH prodomain contains these elements and if so, whether they interact with the type II receptor binding site on the AMH GF.

**Figure 4:**
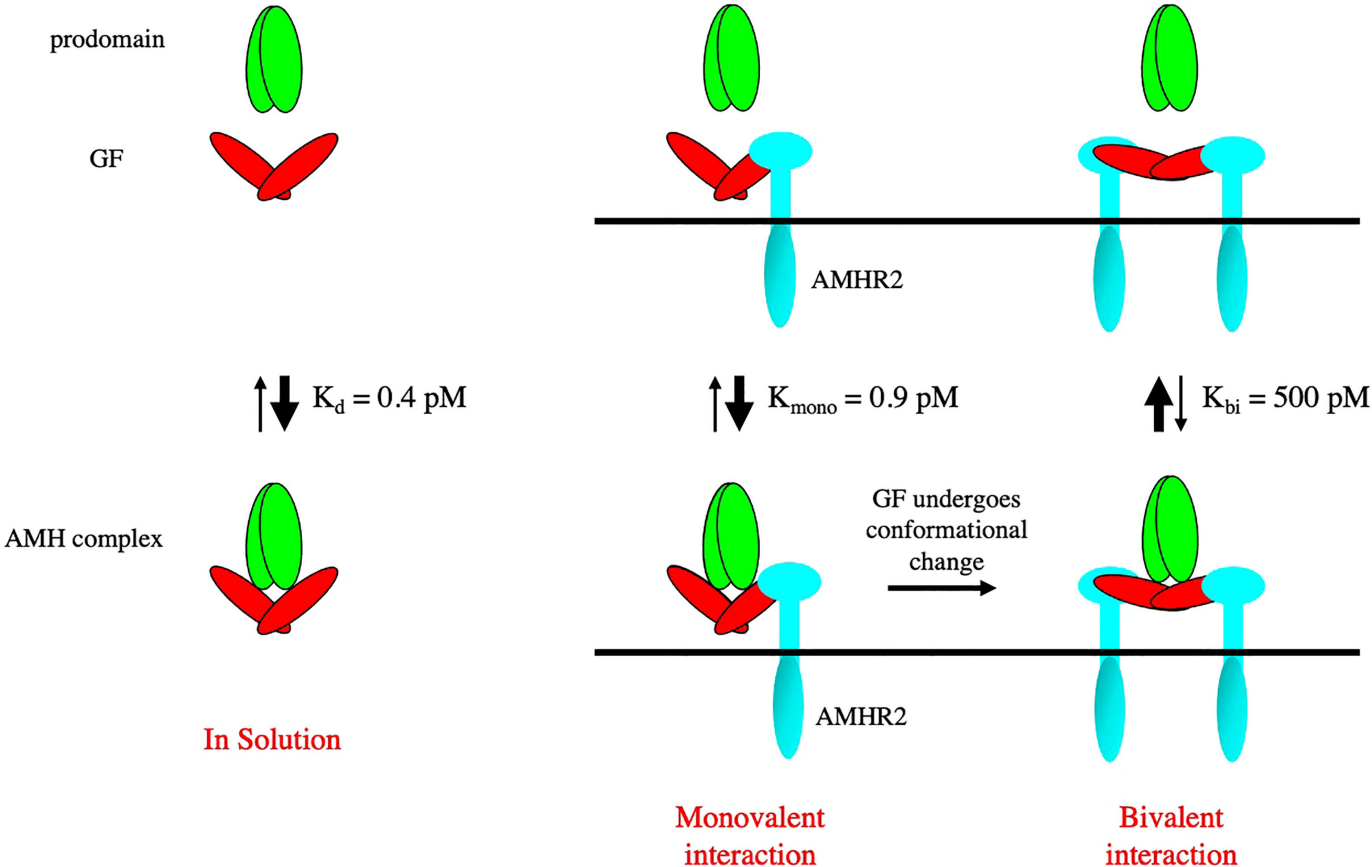
A bivalent interaction of AMHR2 with the AMH noncovalent complex leads to a conformational change in the GF and displacement of the prodomain. The dissociation constants of the AMH complex bound monovalently and bivalently to AMHR2 are shown. Bivalent binding causes a 1000-fold increase in the K_d_ of the complex leading to rapid dissociation.

The AMH noncovalent complex is biologically active (28, 55) and can bind to AMHR2 (56). However, when the AMHR2 is on a surface (as a fusion protein with IgG) or on the surface of cells, the prodomain is displaced after binding to AMHR2 (56). The prodomain of the BMP-7 complex has been shown to be displaced by the type II receptor BMPR2 (57), while the prodomain of the BMP-9 complex has been shown to be displaced by the type I receptor ALK1 (54, 58). When ELISA assays were used to measure the dependence of prodomain displacement on AMH concentration, it was found that prodomain displacement only occurred at low concentrations of the AMH complex, indicating the AMH complex can bind to surface captured AMHR2 in two different states, with one being susceptible to dissociation (59). Subsequently it was shown that when the AMH complex is bivalently bound by two AMHR2 molecules, the dissociation constant for the AMH complex is increased by 1000-fold, compared to the unbound state. Monovalent binding to one AMHR2 molecule had a minimal effect on the dissociation constant, indicating that the receptor binding site in the AMH complex is fully accessible to AMHR2. Furthermore, displacement did not occur when the AMHR2 was in solution, indicating that displacement of the prodomain is caused by a conformational change in the growth factor induced by bivalent binding to AMHR2 on a surface (59). These results are summarized in **Figure 4**. In **Figure 3**, the non-latent complexes of BMP-9 and AMH are compared.

### Interactions of the AMH GF with AMHR2

Due to the limited number of type II receptors, many TGF-β receptors bind multiple ligands. In contrast, AMH and AMHR2 have a monogamous relationship (16, 41). The crystal structure of AMH bound to AMHR2 has provided insights into the reasons for this specificity (29) (**Figure 2C**). The binding sites for AMHR2 on the GF are shown in **Figure 2A**; some of the residues in contact with the GF on AMHR2 are shown in **Figure 2B**. For a detailed description of the interactions between AMH and AMHR2, readers are referred to the chapter in this edition by Thomas B. Thompson and co-authors. Previous mutational studies have also provided insight into how AMH interacts with AMHR2 (31, 42, 60). Of note, the Q496H mutation in the pre-helix loop of the GF may disrupt binding of the type I receptor (31).

### Binding of Type I receptors to the AMH/AMHR2 complex

After the AMH complex binds bivalently to AMHR2, the GF undergoes a conformational change which leads to the displacement of the prodomain as described above. Presumably, it is at this stage that the low affinity type I receptors are recruited. Several observations support this notion. It has been proposed that binding of activin by two ActR2 chains immobilize it in a type I receptor binding-competent orientation (61, 62). BMP-7 requires a cooperative interaction with ActR2 to bind ALK2, which does not rely on receptor-receptor contacts (63). Furthermore, it has been shown that the BMP-2 homodimer (64) and a BMP-2/6 heterodimer (65) must have two functional type II binding sites to activate their receptors and stimulate downstream signaling on cells. Together, these observations suggest that the conformational change induced in the AMH GF upon bivalent binding to AMHR2 is required for the recruitment of the type I receptor. However, without further experimentation, it cannot be ruled out that recruitment of the type I receptor also involves a cooperative interaction with AMHR2, perhaps mediated by their cytoplasmic domains.

To date, there have been few reports in the literature of direct binding of any type I receptor to the AMH/AMHR2 complex. In one study, co-immunoprecipitation assays indicated that ALK-6 was the only type I receptor that could interact with AMHR2 in cells in a ligand dependent manner (66), although subsequent analyses suggested that ALK-6 acts as a negative regulator of intracellular signaling (67). Evidence supporting ALK2 as a type I receptor has come from experiments which measure downstream signaling in cells facilitated by different type I receptors. Assays using a luciferase reporter indicated that ALK2, but not ALK3 could enhance the AMH signaling response (68), while dominant negative variants of ALK2 could attenuate the AMH induced activation of a reporter gene (68, 69). In a Sertoli cell line, AMH signaling was attenuated by a dominant negative variant of ALK3 and by small interfering RNAs (67). ALK2 could compensate for the absence of ALK3 and act in synergy with ALK3 at high concentrations of AMH. It has been reported that in certain cases, ALK2 and ALK3 can bind to the same BMP-2 or BMP-6 homodimer (70). A ligand independent association of ALK2 with AMHR2 was observed in COS cells transfected with the two receptors (71).

Targeted gene disruption experiments in mice have provided the most compelling evidence for the involvement of ALK2 and ALK3 in the regression of the MD mediated by AMH. Because mice with mutations in *Alk2* and *Alk3* are not viable, conditional knockouts were generated using the *cre/loxP* system. A Cre recombinase expression cassette was targeted into the *Amhr2* locus in order to drive expression of the Cre recombinase in the mesenchymal cells of the MD (72). The *Amhr2-cre* mice were then crossed with mice carrying conditional null alleles of *Alk2* or *Alk3* (i.e. LoxP sites were inserted on both sides of the genes making them susceptible to removal by the Cre recombinase in the cells where it is expressed). Conditional inactivation of *Alk3* was found to block MD regression in ~55% of the males (73), whereas conditional inactivation of *Alk2* did not block MD regression in any of the males. When both *Alk2* and *Alk3* were conditionally inactivated, MD regression was blocked in 100% of the males. These results indicated that *Alk3* is the primary type I receptor for AMH induced MD regression, but *Alk2* in the absence of *Alk3* can transduce the AMH signal (73). Mice with mutations in *Alk6* are viable and males do not have retained MDs, indicating that *Alk6* is not essential for AMH induced regression of the MD (69, 74).

**Figure 5:**
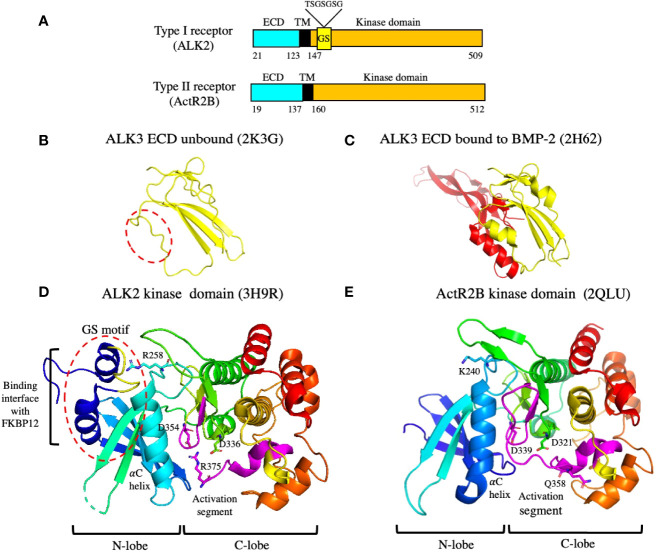
Organization and structures of the type I and type II receptors. **(A)** A schematic diagram showing the organization of the type I and II receptors. The type I receptor contains a GS motif that is phosphorylated by the type II receptor on serine residues, leading to its activation. The amino acid sequence of the GS loop containing the serine residues that are phosphorylated is shown above the GS motif. The numbering corresponds to amino acid residues in UniProtKB accession numbers Q04771 (human ALK2) and Q13705 (human ActR2B). ECD: extracellular domain; TM: transmembrane. **(B, C)** Structures of the ALK3 ECD in solution **(B)** and bound to the BMP-2 ECD **(C)**. The dashed red oval shows that the alpha helix in ALK3 involved in binding BMP-2 is not present in the unbound state. **(D, E)** Structures of an inactive type I receptor kinase domain (ALK2) **(D)** and an active type II receptor kinase domain (ActR2B) **(E)**. The GS motif in ALK2 is indicated by the red dashed oval; it interacts with FKBP12 as shown. The GS motif is composed of two alpha helices connected by a loop (yellow) which contain the serine residues that are phosphorylated by the type II receptor. ALK2 contains a salt bridge between residue R375 and residues D336 and D354. Both aspartate residues are required in all kinases for Mg-ATP binding and catalysis. PDB files used to generate the structures are indicated.

The overall structures of the type I and II receptors are depicted in the schematic diagram shown in **Figure 5A**. All type I and II receptors share sequence and structural homology in both their ECD and kinase domains, indicating that they arose from a common ancestor (14). **Figure 3** shows how type I receptors bind to the TGF-β1and BMP-9 GFs and reveals that their binding modes are somewhat different. In the case of TGF-β1, the type I and II receptor ECDs make contact (75), while in the case of BMP-9, they do not (76). Following TGFR2 binding to TGF-β3 or TGF-β1, the type I receptor binds tightly to the TGF-β/TGFR2 composite surface (75, 77). This may explain why with the TGF-βs, one pair of bound type I and II receptors can signal independent of the other pair (78), while in the case of BMPs, two type II receptors are required for signaling (64, 65). Thus, the TGF-βs use a cooperative model for assembling the signaling complex, whereas several BMPs, activin, and AMH appear to follow an allosteric model, in which bivalent binding to the type II receptor results in a conformational change in the GF and the exposure of the binding site for the type I receptor. In the latter case, cooperative effects could also be involved in the recruitment of the type I receptor.

The ALK3 ECD undergoes a conformational change after binding to ligand. The structures of ALK3 ECD unbound (79) and bound to BMP-2 (80) are shown in **Figures 5B, C**, respectively, revealing that the α-helix in ALK3 is not present in the unbound state. This α-helix makes important contacts with the ligand, indicating that ALK3 uses an induced fit mechanism to adapt to the binding interface in BMP-2. The flexibility of ALK3 may explain how it can bind to so many different ligands including AMH.

### Activation of Type I receptors

The structures of the kinase domains of the type I receptor ALK2 (81) and the type II receptor ActR2B (82) are shown in **Figures 5D, E**. The structures look similar, with both kinases containing an N-lobe, consisting mostly of a five stranded β-sheet, and a C-lobe that is mostly α-helical. However, the ALK2 kinase is in an inactive conformation, whereas the ActR2B kinase is in an active conformation. One major difference is the presence of the GS motif in the type I receptor located just inside the membrane (**Figure 5A**). The GS motif is indicated by the red dashed circle on the ALK2 structure in **Figure 5D** and is composed of two α-helices connected by a loop (colored in yellow). This loop contains several serine residues which are phosphorylated by the type II receptor kinase resulting in its activation. One of the α-helices in the GS motif interacts with the inhibitory protein FKBP12, which has been shown to bind to and stabilize the inactive conformations of several type I receptors (83, 84). In this inactive conformation of ALK2, certain elements critical for catalytic activity are not optimally positioned (81). The GS loop is protected from phosphorylation by residue R258 which buries the GS loop in the kinase N-lobe between the αC helix and β4 strand; the correct positioning of the αC helix is critical for kinase activation. In addition, the inactive conformation is stabilized by the formation of salt bridges between residue R375 in the activation segment and residues D336 (in the catalytic HRD motif) and D354 (in the DFG motif). Both aspartate residues are required in all kinases for Mg-ATP binding and catalysis. These salt bridges are not formed in the active type II kinase domain of ActR2 (**Figure 5E**).

Bringing the inactive type I receptor close to the active type II receptor through interactions of their ECDs with ligand, allows phosphorylation of the serine residues in the GS loop. Phosphorylation of the GS loop in ALK5 results in much higher specificity of the type I receptor kinase for the C-terminal serine residues of Smad2 (see below) and prevents binding to FKBP12 (85). Exactly how the type II kinase phosphorylates the serine residues in the GS loop is not known, since there are no structural studies to date that provide insight into the organization of the intracellular domains in an active receptor complex containing two type I and two type II receptors. Recently, it has been reported that ALK2 and BMPR2 form a heterodimeric complex *via* their C-terminal lobes that is essential for ligand induced receptor signaling (86).

### Phosphorylation of Smads

**Figure 6:**
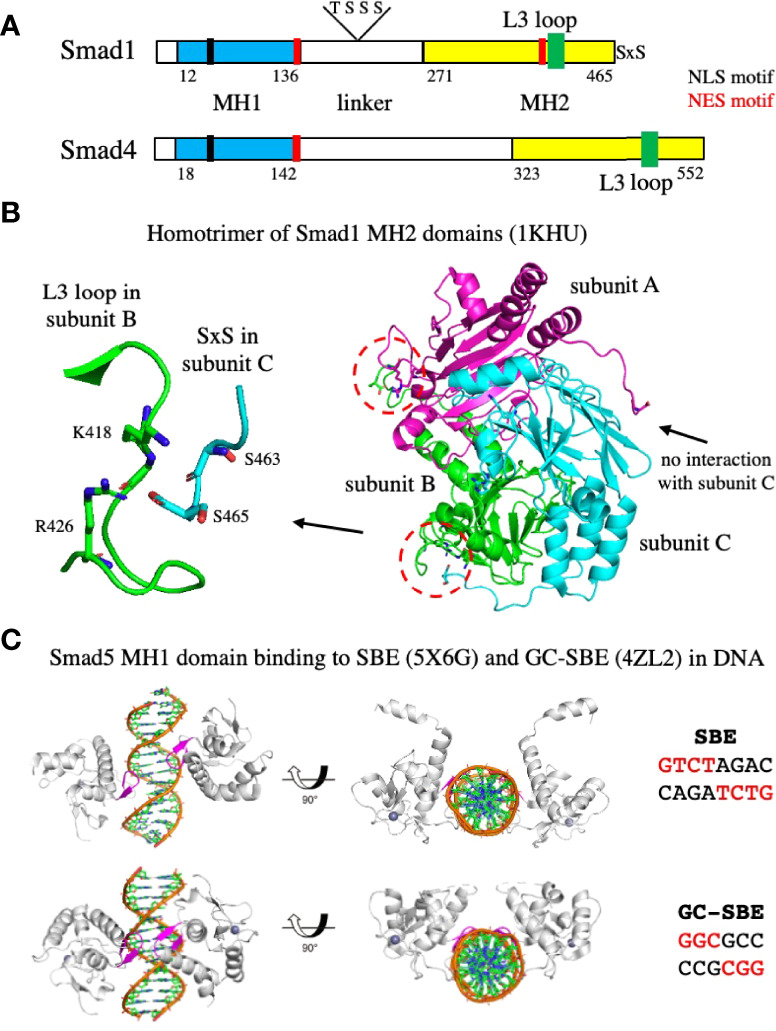
Organization of Smads, homotrimerization of the MH2 domain, and binding of the MH1 domain to DNA. **(A)** A schematic diagram showing the organization of Smad1 and Smad4. Both Smads contain an MH1 domain, a central proline-rich linker, and a carboxy-terminal MH2 domain. Smad1 contains two serine residues at the C-terminus that are phosphorylated by the type I receptor kinase. NLS and NES motifs important in the export into and out of the nucleus, and threonine and serine residues phosphorylated by CDK8/9 (S206 and S214) or GSK-3 (T202 and S210) in the linker of Smad1 are shown. The numbering corresponds to amino acid residues in UniProtKB accession numbers Q15797 (human Smad1) and Q13485 (human Smad4). **(B)** Structure of the Smad1 MH2 domain homotrimer. The C-termini of subunits B and C interact with the L3 loops in subunits A and B, respectively, indicated by the red dashed circles. An enlargement of the interaction between the serine residues at the C-termini of subunit C with residues in the L3 loop of subunit B is shown on the left. The C-termini of subunit A does not interact with the L3 loop of subunit C. **(C)** Structures of the Smad5 MH1 domain bound to the SBE or GC-SBE elements in DNA. The β-hairpin responsible for DNA sequence recognition (shown in magenta) is embedded in the major groove of DNA. PDB files used to generate the structures are indicated.

All Smads share a similar structural organization with an amino-terminal Mad-homology 1 (MH1) domain, a central proline-rich linker, and a carboxy-terminal MH2 domain (**Figure 6A**) (15). The two groups of R-Smads, Smad2 and 3 and Smad1, 5, and 8, share 84-90% identity within the groups (Smad2 and 3 compared or Smad1, 5, and 8 compared), and 56-60% identity between groups (Smad2 and 3 compared to Smad1, 5, and 8). The MH1 domain contains a β-hairpin structure that mediates the binding of Smads to DNA, while the MH2 domain mediates the association with Smad4 and subsequently with other transcription factors after nuclear translocation. Smad activation is dependent on the type I receptors that phosphorylate a common Ser-X- Ser motif present at the carboxyl terminus of the MH2 of R-Smads (13). The TGF-β and activin-specific receptors phosphorylate Smad2 and 3, whereas the BMP-specific receptors phosphorylate Smad1, 5, and 8. AMH has been shown to induce phosphorylation of Smad1, 5, and 8 in all of its target organs (66, 68, 69), as well as induce an interaction with Smad4 and nuclear translocation (66). The *Amhr2-cre* mice have also been used to conditionally knockout the *Smad1, 5, and 8* genes in the mesenchymal cells of the MDs in mice. The results indicated that *Smad5* is the major R-Smad used in AMH-induced MD regression, but *Smad1* and *Smad8* are both capable of replacing *Smad5* (73).

At present there are no structural studies that inform explicitly on how the type I receptors interact with Smads. Anchor proteins that bind to phosphatidylinositol 3-phosphate and recruit Smads to the inner membrane and position them adjacent to activated type I receptors have been identified for Smad2/3 (SARA) (15) and for Smad1 (endofin) (87). For ALK5, it has been proposed that phosphorylation of the GS loop of the kinase domain by the type II receptor, switches the GS region from a binding site for the inhibitory protein FKBP12 into a recruitment site for the R-Smad substrate (85). Mutational studies have identified four residues within the β4-β5 loop of the type I receptor kinase domain that may determine Smad selectivity; an exchange of these residues between BMP and TGF-β type I receptors results in their Smad selectivity being reversed (13). There are some leads as to how the type I receptor and Smad interact to allow phosphorylation, but nothing is conclusive. Ultimately, the carboxy-terminal SxS motif of the R-Smad must be engaged by the substrate pocket in the C-lobe of the kinase domain for phosphorylation to occur (13).

Phosphorylation of the C-terminal serine residues induces trimerization of the R-Smads. Biochemical analyses have indicated that Smad2 and Smad3 exist as monomers *in vivo* and form homoligomers upon phosphorylation of the SxS motif by ALK5 (88, 89). Oligomerization was found to occur in the absence of Smad4 and in the case of Smad2, the analysis showed that it was most likely a homotrimer. The crystal structure of the phosphorylated Smad2 MH2 domain revealed the formation of a homotrimer mediated by the C-terminal phosphoserine residues (89). Similar results were observed with Smad1. When the two serine residues at the C-terminus of Smad1 were mutated to aspartic acid to mimic the structural and electrostatic properties of phosphorylation, the modified Smad1 protein superactivated a Smad1/Smad4 dependent signaling response and underwent a monomer to trimer transition at high concentration (90). The X-ray structure of an unphosphorylated Smad1 MH2 domain revealed that it forms an asymmetric trimer (90), shown in **Figure 6B**. Two distinct structural arrangements of the C-terminal tails are apparent: The tails of subunits B and C interact with the L3 loops of subunits A and B, respectively, while the tail of subunit A rotates in a different direction, leaving the L3 loop of subunit C unoccupied. A blowup of the interaction between the tail of subunit C and the L3 loop of subunit B is shown on the left side of **Figure 6B**. The proximity of residue S465 at the C-terminus to residues K418 and R426 in the L3 loop, suggest that phosphorylation of S465 will generate electrostatic interactions between the phosphoryl group and the positively charged side chains of residues K418 and R426 that will strengthen subunit to subunit contact. Phosphorylation of S463 also introduces favorable electrostatic and H-bond interactions with residues in the L3 loop (90). Thus, phosphorylation of the C-terminal serine residues is likely to promote trimerization of Smad1.

The R-Smad homotrimers are subsequently converted to R-Smad/Smad4 heterotrimers. The absence of an interaction between the C-terminal tail of subunit A with the L3 loop of subunit C (**Figure 6B**) suggests that the homotrimeric interactions may be less than optimal, allowing preferential formation of the Smad1/Smad4 heterotrimer. Structural modeling of a 2 to 1 Smad1/Smad4 heterotrimer has indicated that the Smad4 subunit would be favorably tilted toward the Smad1 subunit (90). Activation of Smad2 in the presence of Smad4 prevented Smad2 homo-oligomerization, providing biochemical evidence that Smad2 can form heterotrimers with Smad4 (88). The crystal structures of the Smad2/Smad4 and Smad3/Smad4 complexes are heterotrimers, comprising two phosphorylated R-Smad subunits and one Smad4 subunit (91).

### Nuclear translocation of Smads

After the formation of the R-Smad/Smad4 heterotrimer, it translocates to the nucleus to regulate target genes. Nuclear localization of Smads is controlled by two opposing signals: The nuclear localization signal (NLS) and the nuclear export signal (NES). The NLS motif is a single stretch of 5-6 positively charged amino acids that mediates the transport of proteins into the nucleus by docking with importin-α and -β at the cytoplasmic side of the nuclear pore (92). The NES motif is a short stretch of critically spaced hydrophobic residues, usually leucines (LXXLXXLXL), that conveys the protein out of the nucleus (93) and is mediated by exportin-1, also known as CMR1 (94). Smad1 contains an NLS motif in the MH1 domain (95) and two NES motifs, one in the MH1 domain (96) and one in the MH2 domain (95) (**Figure 6A**). Mutations in either NES motif convert Smad1 into an exclusively nuclear location. All motifs are required for optimal transcriptional activation by Smad1, indicating that it is under constant nucleocytoplasmic shuttling, and that nuclear accumulation is the result of a change in the balance of NLS and NES opposing signals (95). At one point it was thought that Smad4 did not have an NLS motif and was brought into the nucleus through its interaction with the R-Smads. However, Smad4 was shown to contain an NLS motif at a similar location as Smad1, that requires additional basic residues on the C-terminal side up to 86 amino acids away to confer nuclear localization (97). Smad4 contains an NES signal which overlaps with the NES2 motif in Smad 1, that is required for optimal transcriptional activation and is inactivated by TGF-β induced hetero-oligomerization with R-Smads (98). Conformational changes within MH1 and MH2 domains and associations between domains can either expose or mask the motifs and affect their functionality.

R-Smad–Smad4 complexes in the nucleus are further phosphorylated in the linker region by other kinases that induce interaction with additional transcription factors and subsequently with ubiquitin ligases (99) (**Figure 6A**). Phosphorylation of Smad1 by CDK8/9 at positions S206 and S214 creates docking sites for YAP, another transcriptional effector required for certain BMP actions (100). Phosphorylation at the CDK8/9 sites also trigger the subsequent phosphorylation at T202 and S210 by GSK-3, which regulates the duration of the activated pSmad1 signal by switching the docking site preference from YAP to the ubiquitin ligase Smurf1, resulting in its degradation (101). Thus, nuclear CDK8/9 participates in a cycle of Smad activation and disposal that is critical for optimal BMP signaling (100).

### Binding of Smads to DNA

After translocation to the nucleus, the phosphorylated heterotrimeric Smad complexes are selectively recruited to target genes. The MH1 domains of Smad2/3-Smad4 complexes mostly bind to the GTCTAGAC site (referred to as the Smad binding element, SBE) (102). In contrast, the best-defined Smad1/5-binding site is GGCGCC (referred to as the GC-SBE) (103) and is present in the enhancers of BMP-responsive genes, such as the ID and VENTX genes (104–106). Crystal structures have been solved for the MH1 domains of Smad1 (107), Smad3 (102), and Smad4 (108) bound to the SBE site. Two MH1 domains are bound to the motif, with each MH1 domain binding on opposite sides of the DNA to a half site of the SBE (GTCT) (102). In all three MH1 domains, an 11-residue β-hairpin, responsible for DNA sequence recognition, is embedded in the major groove of DNA (99), and the amino acids that make contacts with the bases in the DNA are conserved. The structures of the Smad5 MH1 domain bound to the SBE and the GC-SBE have also been determined (109) (shown in **Figure 6C**). The Smad5 MH1 domain contacts the SBE and the GC-SBE with the same β-hairpin (shown in magenta) and the same conserved amino acids. However, due to the shorter GC-SBE motif, the two MH1 domains are closer together than on the SBE motif. In addition, binding of the Smad5 MH1 domain induces different DNA conformations in the SBE and GC-SBE sequences (109).

The affinity of Smads for DNA is weak (K_d_ = 0.1 μM) (102), which may explain why multiple SBEs are needed to get transcriptional activation in cell culture. Although some MH1 domains do not show cooperative binding to palindromic SBEs, full length Smads would be expected to behave differently. As described above, MH2 domains undergo heterotrimerization, so MH1 domains as part of a trimeric Smad complex should be capable of binding with higher affinities than single MH1 domains to the SBE or GC-SBE sites, especially if multiple sites are present in the promoter or enhancer (110). Higher-affinity interactions should also be promoted by the recruitment of additional transcription factors that bind to the MH2 domains and bind to different DNA sequences. The initiation of Smad dependent gene transcription involves the assembly of a preinitiation complex containing general transcription factors and RNA polymerase II, and its correct positioning at the transcription start site of a target gene. This complex is then regulated by Smads and their associated transcription factors bound at proximal promoter regions or at more distal enhancers (103). The cooperating transcription factors can be either activators or repressors. An important transcription factor that interacts with Smad1/5 is RUNX2, which mediate the activation of genes involved in osteoblast formation, such as osteocalcin, osteopontin, collagen alpha-1(X) chain, and Smad6 (103). Smad1 has also been shown to interact with HoxC8, Nkx3-2, YY1, and GATA factors 4, 5, and 6 (110), as well the myeloid lineage regulator CEBPA and the erythroid regulators GATA factors 1 and 2 (111). Recently a crystal structure of the Smad2 MH2 homotrimer in complex with a domain of the transcriptional activator CBP was reported, showing that CBP forms an amphiphilic helix on the hydrophobic surface of Smad2 (112).

## Network of Signaling Pathways Involved in Müllerian Duct Regression

**Figure 7:**
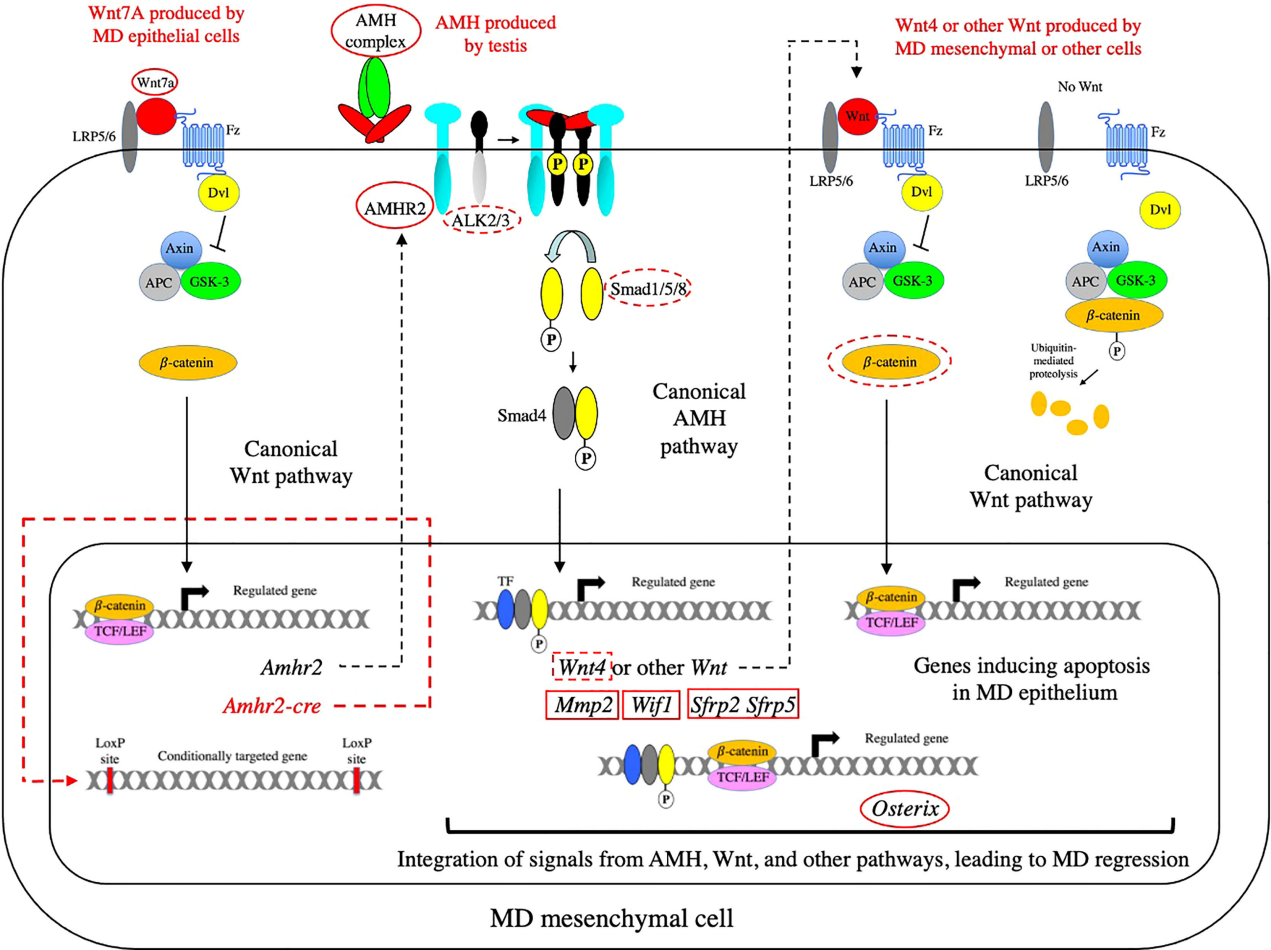
The network of signaling pathways required for MD regression. The AMH canonical signaling pathway is shown in relation to the upstream and downstream Wnt canonical pathways that are required for MD regression. The canonical Wnt pathway on the right of the diagram shows how catenin is degraded in the absence of a Wnt signal. In the absence of Wnt signaling, TCF factors associate with Groucho family proteins to repress transcription (not shown). When Wnt binds to its receptors (Fz and LRP5/6), Disheveled (Dvl) is recruited and activated, resulting in the inhibition of the serine/threonine kinase, GSK-3, and leading to the accumulation of β-catenin and its translocation to the nucleus. In the nucleus, β-catenin displaces Groucho to bind TCF/LEF and activate transcription. Genes or proteins that affect MD regression as null mutations or conditional knockouts are indicated with red solid or dashed ovals, respectively. Genes that do not affect MD regression as null mutations or conditional knockouts are indicated with red solid or dashed rectangles, respectively. β-catenin is not circled in the Wnt7a pathway shown on the left because the conditional knockout of β-catenin did not affect expression of AMHR2. Since both *Amhr2* and the *Cre-recombinase* (under the control of the *Amhr2* promoter) would be induced at the same time, there is almost certainly a time delay between establishment of a competent AMH signaling pathway and the inactivation of β*-*catenin in the Wnt7a pathway required for the induction of AMHR2. Fz, Frizzled; LRP5/6, LDL-receptor-related protein 5/6; APC, adenomatous polyposis coli protein.

The signaling pathways that have been implicated in MD regression are depicted in the schematic diagram shown in **Figure 7**. Below, each of these pathways are discussed (going from left to right) with a goal of showing how they are integrated to induce regression. Much of this is based on the work of Richard R. Behringer and his colleagues at the University of Texas (113).

### The Wnt7A pathway is necessary for expression of AMHR2

Wnt7a is expressed in the MD epithelium and is required for MD regression during male sexual differentiation. Male mice lacking Wnt7a have retained MDs due to the absence of AMHR2 (114). Wnt7a belongs to the Wnt family of ligands which play an important role in the development and differentiation of many cell types during development and adult homeostasis. In the canonical Wnt pathway, Wnts signal by binding to seven-pass transmembrane receptors, Frizzled (Fz), which results in the inhibition of the serine/threonine kinase, GSK-3, mediated by Disheveled (Dvl). This leads to the accumulation β-catenin and translocation to the nucleus where it associates with TCF/LEF and activates transcription (115) (See **Figure 7**). Wnt signaling can also be mediated by two other pathways: The planar cell polarity pathway which regulates the cytoskeleton, or the calcium pathway which regulates calcium inside the cell (115). Wnt7a has been shown to signal through the canonical pathway in a number of oncological models (116, 117) and in synapse formation (118). There is evidence that Wnt7a induction of AMHR2 is also mediated by the canonical pathway. A luciferase reporter gene driven by the *AMHR2* promoter was activated by β-catenin and the activation was dependent on TCF4 binding sites in the AMHR2 promoter (119). Both *Wnt7a* (114) and β-catenin (120) are required for female MD differentiation indicating that β-catenin is a downstream effector of Wnt7a for this function.

### Genes induced *via* the AMH canonical pathway

Several genes have been identified that are induced *via* the AMH canonical pathway. *Wnt4* is expressed in a sexually dimorphic pattern during the period of MD regression. *Wnt4* starts to be expressed at E13.5 in the MD mesenchyme of male mice, but not female mice, and persists in males throughout the period of regression (121). *Wnt4* expression was absent in *Amhr2*-null males, but present in the MD mesenchyme of females ectopically expressing human AMH, indicating that AMH signaling is both required and sufficient for *Wnt4* expression at the onset of MD regression (121). A conditional knockout of Wnt4 using *Amhr2-cre* mice showed that MD regression occurred normally in the absence of *Wnt4*, indicating that it is not required for MD regression.

Expression of a matrix metalloproteinase, Mmp2, is highly upregulated in the MD mesenchyme of male, but not female mice at day E13 (20). In *Amh*-null males, the male-specific activation of Mmp2 was not observed. MD regression was partially blocked in rat urogenital organ culture by protease inhibitors and antisense oligonucleotides to Mmp2, while exogenous Mmp2 triggered apoptosis in the MDs of female urogenital ridges (20). A role for Mmp2 in regression would be consistent with previous studies showing a correlation between MD regression and degradation of the extracellular matrix (21–23). However, a knockout of *Mmp2* in male mice did not affect MD regression, indicating that it is not required for MD regression. Humans with defects in MMP2 display a severe impairment of bone and cartilage development, suggesting that Mmp2 may act redundantly with other Mmp genes in the mouse.

Wnt inhibitory factor 1 (WIF1) is a secreted frizzled-related protein that blocks binding of Wnts to receptors. *In situ* hybridization revealed a pattern of expression of *Wif1* in male mesenchyme similar to *AMHR2*, that was absent in urogenital ridges from females and *Amhr2*-null males (122). Exposure to exogenous AMH induced *Wif1* expression in the MD mesenchyme of female urogenital ridges, while knockdown of *Wif1* expression in male urogenital ridges by *Wif1*-specific siRNAs resulted in MD retention, consistent with WIF1 playing a role in MD regression (122). However, a knockout of *Wif1* in male mice did not affect MD regression, indicating that it is not required for MD regression, or that there is another similar gene that can replace its function. Two members of a different family of Wnt inhibitors, secreted frizzled-related proteins (SFRPs), are also expressed in a sexually dimorphic pattern (123). SFRP2 and SFRP5 proteins are expressed in the mesenchymal cells of the male MD, but not in mice with *Wnt7a* null mutations which lack AMHR2. Mice containing null mutations in both *Sfrp2* and *Sfrp5* exhibit normal MD regression.

### β-catenin is required for MD regression

With several Wnts and Wnt antagonists being induced by AMH in the MD mesenchyme and Wnt7a having an essential role in MD regression, the question was raised whether the Wnt canonical pathway played an essential role in regression. An early insight into this matter was the observation that AMH signaling induces the accumulation of β-catenin in the cytoplasm and the transcription factor, LEF1, in the nucleus of male MD mesenchyme cells (25). Cytoplasmic accumulation of β-catenin was also observed in MD mesenchymal cells of female rat urogenital ridges exposed to AMH, but not in untreated female urogenital ridges. Subsequently, it was shown that conditional inactivation of β-catenin in the MD mesenchyme using *Amhr2-cre* mice, caused MD retention in males, indicating a requirement of β-catenin for MD regression (121). The accumulation of β-catenin in the cytoplasm was significantly reduced and LEF1 upregulation was not observed in the MD mesenchymal cells of the conditional β*-*catenin mutant males at E14.5, confirming that β*-*catenin had been specifically inactivated in these cells. However, at E13.5, both *AMHR2* and *Wnt4* were expressed in the MD mesenchyme of the conditional β*-*catenin mutant males, indicating that AMH signaling was activated in the MD mesenchymal cells in the absence of β-catenin function.

The perturbations observed after the conditional inactivation of β-catenin and the finding that AMH is required for the cytoplasmic accumulation of β-catenin in male MD mesenchymal cells (25) indicate that there is a Wnt/β-catenin pathway required for MD regression which is downstream of the AMH and Wnt7a signaling pathways (121). Although *Wnt4* would be a likely candidate for this Wnt/β-catenin pathway, the conditional knockout of *Wnt4* did not cause MD retention, indicating that either more complete recombination might be required by the Cre-recombinase, perhaps because *Wnt4* encodes a secreted protein, or another Wnt mediates this Wnt/β-catenin pathway (121). Apoptosis of MD epithelial cells requires this downstream Wnt/β-catenin pathway. When β*-*catenin was inactivated in males, the level of cleaved caspase-3 positive in the MD epithelium was significantly reduced, indicating that β-catenin is required in MD mesenchymal cells to induce apoptosis in MD epithelial cells (121).

### Integration of AMH, Wnt, and other pathways

The expression of *Osterix (Osx)*, a C2H2-type zinc transcription factor, in male MD mesenchymal cells, is regulated by both of the AMH and Wnt canonical pathways (124). *Osx* was identified using transcriptional profiling methods and shown to have a male specific pattern of expression in MD mesenchymal cells. *Osx* expression was absent in *Amhr2*-null males, but present in the MD mesenchyme of females ectopically expressing human AMH, indicating that AMH signaling is both required and sufficient for *Osx* expression at the onset of MD regression. In mice with β-catenin conditionally inactivated, there was a significant decrease in the level of *Osx* transcripts in the MD mesenchyme, indicating that *Osx* is also regulated by the Wnt/β-catenin pathway. Furthermore, MD regression was delayed in *Osx*-null male mice, demonstrating that *Osx* regulates MD regression (124). In **Figure 7**, the activation of *Osx* is shown by activated Smad and β*-*catenin/TCF complexes binding individually to promoter/enhancer sequences, but it has been shown that BMP can induce an interaction between Smad1 with β-catenin and TCF4 to activate the c-myc promoter (125). Also, Smad3 (126) and Smad4 (127) have been shown to associate with β-catenin and TCF/LEF1 to activate the Xenopus twin promoter. So formally, it is possible that the Smad and β*-*catenin/TCF complexes associate to activate *Osx* transcription.

In hindsight, it is not surprising to find that Wnt and AMH pathways are integrated to bring about regression of the MD, since previous studies have established that Wnt and BMP pathways interact during other biological processes. This interaction can occur in the cytoplasm as effectors of one pathway are modified by effectors from the other pathway, and in the nucleus where the Smad and TCF/LEF transcription factors converge to regulate transcription. During embryonic patterning in *Xenopus*, dorsal-ventral or anterior-posterior cell fates are controlled by gradients of BMPs or Wnts, respectively (128); integration may occur at the level of Smad1 phosphorylation by GSK-3 (101). Both pathways participate in the development (129) and regeneration (111) of the hematopoietic system. Chromatin immunoprecipitation studies have shown that Smad1 and TCF7L2 occupy sites close to other transcriptional regulators in the promoters of hematopoietic genes (111). It is likely that other signaling pathways are involved in MD regression. Overactivation of the hedgehog pathway has been shown to interfere with MD regression, indicating that the AMH and hedgehog signaling pathways probably intersect in places (130).

### Smad independent pathways

Activated receptors of the TGF-β family also signal through non-Smad signaling pathways. This includes the mitogen-activated protein kinase (MAPK) pathways, which comprise the extracellular signal-regulated kinase (ERK), c-Jun amino terminal kinase (JNK), and p38 MAPK families. In addition, the activated receptors can signal *via* IκB kinase (IKK), phosphatidylinositol-3 kinase (PI3K) and Akt, and Rho family GTPases (131, 132). The non-Smad pathways are, in general, activated by phosphorylation of tyrosine residues on either type I or II receptors, and the recruitment of proteins that bind to phosphotryrosine. Tyrosine phosphorylation can result from autophosphorylation or cross phosphorylation of type I and II receptors, or phosphorylation by other tyrosine kinases (131). Although the kinases of TGF-β type I and II receptors mostly phosphorylate serine and threonine residues, they are also capable of phosphorylating tyrosine residues (133). At present, there are only a limited number of reports that AMHR2 can signal *via* Smad independent pathways. AMH was reported to inhibit the androgen dependent growth of a prostate cancer line *via* an NFκB-dependent, but Smad-independent mechanism (134). In another study, blocking AMH signaling in two lung cancer cell lines resulted in lower levels of phosphorylated (and activated) Akt; lower levels of the phosphorylated Akt activator, PDK1, were also observed (135).

Non-Smad signaling pathways are associated with TGF-β receptors that reside in caveolar compartments of the cell membrane (a special type of lipid raft), similar to receptor tyrosine kinases, whereas Smad-mediated signaling pathways are associated with TGF-β receptors that reside in clathrin-dependent endosomal compartments (132). Localization to caveolae can lead to restricted lateral movement and an enrichment of receptors (136). It should also be noted that, in addition to transcriptional regulation, R-Smads have a role in chromatin remodeling required for transcriptional activation and in the processing of microRNAs involved in RNA silencing (15).

## Concluding Remarks

The goal of this review article was to illuminate how AMH interacts with its type II receptor to activate a canonical pathway resulting in the induction of several genes and how the AMH canonical pathway is integrated with a Wnt pathway required for MD regression. However, details in some areas have been limited in the interest of space and comprehension. For more information, the reader is referred to the following reviews (14, 99, 132, 137). One topic that was not covered is how the AMH signals can be distinguished from BMP signals, an issue that is receiving considerable attention. As noted in a recent review article by Nickel and Mueller (138), “It seems illogical that on the one hand Nature has diversified growth factors of this family to more than 30 known members, but at the same time restricted the signaling outcome of all ligands to initiate intracellular signaling pathways in just two different flavors: Smad1/5/8 or Smad2/3”. Part of the answer may lie in understanding how differences in the binding affinities and kinetics of specific ligand-receptor interactions affect the assembly of receptor signaling complexes, and whether distinct receptor signaling complexes can be assembled because of these differences that have distinct signaling properties (138).

## Author Contributions

The author confirms being the sole contributor of this work and has approved it for publication.

## Conflict of Interest

The author declares that the research was conducted in the absence of any commercial or financial relationships that could be construed as a potential conflict of interest.

## Publisher’s Note

All claims expressed in this article are solely those of the authors and do not necessarily represent those of their affiliated organizations, or those of the publisher, the editors and the reviewers. Any product that may be evaluated in this article, or claim that may be made by its manufacturer, is not guaranteed or endorsed by the publisher.
